# COGNITIVE TRAJECTORIES BEFORE AND AFTER SLEEP TREATMENT INITIATION IN U.S. OLDER ADULTS WITH SLEEP DISTURBANCE

**DOI:** 10.1093/geroni/igz038.1499

**Published:** 2019-11-08

**Authors:** Christopher N Kaufmann, Mark W Bondi, James D Murphy, Xin Tu, Alison A Moore

**Affiliations:** 1 Division of Geriatrics and Gerontology, Department of Medicine, University of California San Diego, La Jolla, California, United States; 2 Department of Psychiatry, University of California San Diego, La Jolla, California, United States; 3 Department of Radiation Medicine and Applied Sciences, University of California San Diego, La Jolla, California, United States; 4 Division of Biostatistics, Department of Family Medicine and Public Health, University of California San Diego, La Jolla, California, United States

## Abstract

Sleep disturbances are associated with cognitive decline but it is not clear if initiation of sleep treatments mitigates decline. We used the 2006-2014 Health and Retirement Study. At each wave, participants were administered cognitive assessments and scores were summed (values=0-35; higher=better cognition). All participants also reported if, in the past two weeks, they had taken medications or used other treatments to improve sleep. Our sample (N=4,650) included individuals who at baseline were cognitively normal and untreated for sleep, and at any wave reported some sleep disturbance. We characterized cognitive performance over study period with comparisons before and after sleep treatment initiation. Between 2006-2014, participants exhibited declines in cognitive performance (B=-2.40; 95% CI=-2.73, -2.06; p<0.001) after controlling for confounders. Following sleep treatment, cognitive decline became less pronounced (interaction B=0.94; 95% CI=0.21, 1.67; p=0.013). Results suggest that in older adults with sleep disturbance, initiation of sleep treatment may slow cognitive decline.

